# Locally Advanced Breast Cancer: Treatment Patterns and Predictors of Survival in a Saudi Tertiary Center

**DOI:** 10.7759/cureus.15526

**Published:** 2021-06-08

**Authors:** Nora H Trabulsi, Alaa A Shabkah, Reem Ujaimi, Omar Iskanderani, Mai S Kadi, Nuran Aljabri, Liane Sharbatly, Manal N AlOtaibi, Ali H Farsi, Mohammed O Nassif, Abdulaziz M Saleem, Nouf Y Akeel, Nadim H Malibary, Ali A Samkari

**Affiliations:** 1 Department of Surgery, King Abdulaziz University Faculty of Medicine, Jeddah, SAU; 2 Department of Radiation Oncology, King Abdulaziz University Faculty of Medicine, Jeddah, SAU; 3 Department of Community Medicine, King Abdulaziz University, Jeddah, SAU

**Keywords:** locally advanced, breast cancer, survival, overall survival, relapse, disease-free survival, outcome, saudi arabia

## Abstract

Background

Breast cancer (BC) is the most common cancer in the Kingdom of Saudi Arabia (KSA) and the second leading cause of cancer-related mortality. About 40% of BC in KSA is locally advanced BC (LABC), which has been associated with poorer survival compared with early diagnosed BC.

Objective

To review the presentation and outcomes of LABC, including the characteristics of the disease, different treatment modalities, overall survival (OS), disease-free survival (DFS), and local recurrence in relation to different radiotherapy (RT) techniques.

Methods

We retrospectively reviewed the medical records of 153 female patients with pathologically proven LABC diagnosed at King Abdulaziz University Hospital, Jeddah, KSA, between 2009 and 2017. We obtained data on patient demographics, stage of cancer at diagnosis, tumor characteristics (subtype and receptor status), type of surgery, systemic treatments received (hormonal, targeted therapy, and chemotherapy), RT variables, and recurrence and death dates. Data were analyzed to assess OS and DFS by using Kaplan-Meier analyses and the log-rank test. Univariate and multivariate Cox proportional hazard regression analyses were used to explore and identify factors associated with survival.

Results

The median survival time in the study population was 9.16 years. Older age (65+ years) was associated with worse OS and DFS than was younger age (<65 years) (hazard ratio (HR) 3.20, 95% CI 1.48-6.90, P = 0.003 and HR 2.21, 95% CI 1.12-4.36, P = 0.022, respectively). Regarding the type of surgery, having a mastectomy was associated with worse OS and DFS than was having a lumpectomy (HR 2.44, 95% CI 0.97-6.12, P = 0.05 and HR 2.41, 95% CI 1.13-5.14, P = 0.023, respectively). Positive estrogen and progesterone receptor status was associated with better OS and DFS than was a negative estrogen or progesterone receptor status (HR 0.13, 95% CI 0.05-0.30, P < 0.001 and HR 0.21, 95% CI 0.11-0.41, P < 0.001, respectively). Patients who received RT had a lower risk of recurrence than did those who did not receive RT (P = 0.011). Moreover, three-dimensional conformal RT was associated with lower local recurrence than intensity-modulated RT or volumetric-modulated arc therapy (P = 0.003).

Conclusion

Multiple factors can affect the OS and DFS in LABC. Younger patients, having hormone-positive disease, and undergoing lumpectomy were associated with better outcomes. Adjuvant RT may improve local control and the use of three-dimensional conformal RT was superior for local control. Prospective studies with larger sample sizes are needed to further highlight these findings and to assess the role of chemotherapy and targeted therapy in patients with LABC.

## Introduction

In 2020, there were 2,261,419 new cases of breast cancer (BC) worldwide, accounting for 11.7% of all types of cancer. BC is the fifth leading cause of cancer-related mortality, comprising 6.9% of cancer-related deaths [1].

In the Kingdom of Saudi Arabia (KSA), BC is the most common cancer, accounting for 16.7% of all cancer cases and 30% of all cancer cases among Saudi women. Moreover, it is the second leading cause of cancer-related mortality in KSA, accounting for 8.4% of cancer-related deaths [1]. About 40% of BC is locally advanced BC (LABC) at the time of diagnosis [2].

LABC is an unusual presentation among women in the United States and Europe, as it accounts for only 4%-8% of cases of BC, whereas LABC is more common in KSA [2,3]. This could be attributed to multiple factors, including the absence of a national screening program in KSA and difficult access to healthcare facilities [4]. Even though LABC is not common in Western countries, most of the available studies in the literature that have explored different aspects of LABC have been conducted in Western countries, with a scarce number being performed in countries in the Middle East, including KSA.

Given its advanced presentation, LABC is associated with poor survival. The median survival of LABC across previous studies ranged between 28 and 66 months, and the five-year overall survival (OS) is reported to be between 40% and 75% [5-8]. Disease-free survival (DFS) is reported to be around 67% in three years [9] and the five-year locoregional recurrence rate (LRR) is 7%-9% [10,11].

Neoadjuvant and adjuvant therapies are required in addition to surgery to reduce the LRR [12]. Adjuvant radiotherapy (RT) has been shown to improve locoregional control in LABC [13], but the influence of different RT techniques on the LRR has not been well studied.

The aim of our research was therefore to review the presentation and outcomes of LABC at a major hospital in KSA in relation to different RT techniques, including the characteristics of the disease, different treatment modalities, OS, DFS, and local recurrence.

## Materials and methods

Study design and population

In this study, we examined a cohort of 153 patients who were diagnosed with LABC in King Abdulaziz University Hospital (KAUH) between 2009 and 2017 and were followed up to January 2020 to assess outcome. KAUH is a major university hospital and a tertiary care center in Jeddah, Saudi Arabia, that serves as one of the cancer referral centers. Approval for this study was granted by the Research Committee of the Unit of Biomedical Ethics at KAUH (Reference number 464-17).

Data collection and endpoints

We performed a retrospective review of the medical records of female patients with pathologically proven LABC diagnosed at KAUH between 2009 and 2017. LABC was defined on the basis of the tumor-node-metastasis (TNM) pathological staging system, according to the American Joint Committee on Cancer [14], as any patient who presented with stage III disease (T3N1, T4, inflammatory BC, or N2-N3 disease). Patients with early BC (stage I-II) or metastatic disease (stage IV) were excluded.

For each patient, we obtained demographics, stage of cancer at the time of diagnosis (TNM), tumor characteristics (subtype and receptor status), type of surgery, systemic treatments received (hormonal, targeted therapy, and chemotherapy), RT variables, and recurrence and death dates. The primary endpoints were the OS and DFS rates and the factors associated with them. The secondary endpoint was local recurrence in relation to different RT techniques.

Data analysis

Statistical analysis was performed with R v 3.6.2 (R Studio, version 3.5.2, Boston, MA, USA). Data were summarized as mean ± standard deviation for continuous variables and as counts (percentages) for categorical variables. Survival analysis was used to model the time to event (death) since the diagnosis of LABC. Survival time was calculated for each patient. Surviving patients were censored at the date of the last follow-up.

The Kaplan-Meier estimator was used to compare survival across groups and the log-rank test to compare survival between groups. The maximally selected rank statistics were used to identify a cut-off point for age. Univariate Cox proportional hazard (CPH) regression analysis was used to explore factors associated with survival. Significant predictors (identified in univariate CPH) were used in a multivariate CPH model to derive a model that can predict survival. The predictive power of the model was assessed by using index-corrected Somers’ D (Dxy). The adjusted hazard ratio (HR) and associated 95% confidence interval (CI) were calculated for each of the predictors in the final model. Hypothesis testing was performed at a 5% level of significance.

A nomogram was developed on the basis of the final multivariate CPH model. Points corresponding to the value of each predictor were calculated by using the nomogram and added to develop a total score; the predicted five-year survival probability (≥ 5 years) was calculated based on the overall score. Model validation was performed by using 1000 bootstrapped samples. The predictive power of the model was assessed with index-corrected Somers’ D (Dxy).

## Results

Descriptive statistics

Patient demographics are shown in Table 1. A total of 153 patients who were diagnosed with LABC in our institute were enrolled in this study. Their mean age was 54.3 years (SD 12.3). Regarding pathological classification, 80% of LABC was invasive ductal BC and13% invasive lobular BC. Around 42% of patients had T3/4 tumors and 43% had a lymph node status of N2/3. The majority of patients (59.5%) were diagnosed with estrogen (ER)+/progesterone (PR)+ (PP) tumors, 77.8% of the patients did not express the human epidermal growth factor receptor type 2 (HER2) gene (HER2-), and 17.95% had triple-negative tumors (ER-/PR- (NN), HER2-).

**Table 1:** Descriptive statistics of patients with locally advanced breast cancer included in the study. ER: estrogen receptor; PR, progesterone receptor. *ER and PR status were combined as both negative (NN), both positive (PP), or only one positive (NP).

Characteristic	N = 153
Age (SD)	54.3 (12.3)
Diagnosis	
Ductal	122 (79.7%)
Lobular	20 (13.1%)
Other	11 (7.19%)
T stage	
T1/2	88 (57.5%)
T3/4	65 (42.5%)
N stage	
N0/1	87 (56.9%)
N2/3	66 (43.1%)
ER/PR status*	
NN	42 (27.5%)
NP	20 (13.1%)
PP	91 (59.5%)
HER2 status	
Negative	119 (77.8%)
Positive	34 (22.2%)
Targeted therapy	
No	124 (81.0%)
Yes	29 (19.0%)
Radiotherapy	
No	32 (20.9%)
Yes	121 (79.1%)
Chemotherapy	
None	21 (13.7%)
Adjuvant	81 (52.9%)
Neoadjuvant	51 (33.3%)
Type of breast surgery	
Lumpectomy	45 (29.4%)
Mastectomy	108 (70.6%)

Almost 33% of the patients received neoadjuvant chemotherapy and 53% received adjuvant chemotherapy. The majority (81%) did not receive targeted therapy, but most (79.1%) received adjuvant RT. Mastectomy was performed in 70.6% of the patients. The median survival time in the study population was 9.16 years.

Overall survival

Univariate Analysis

**Figure 1:**
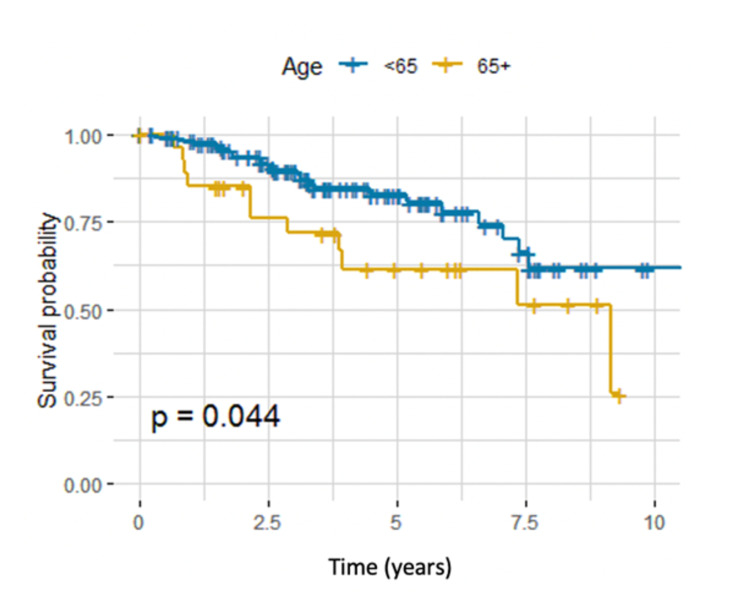
Kaplan-Meier estimator for age groups, log-rank test (P = 0.044).

Our data showed that age, receptor status, and type of surgery were important predictors of OS, each identified by using univariate CPH regression to estimate the risk of death in patients with LABC. Patients who were 65 years or older at the time of diagnosis were almost twice as likely to die as patients who were <65 years (HR 2.08, 95% CI 1.00-4.31, P = 0.049) (Table 2). Survival curves across different age groups are shown in Figure 1.

**Table 2:** Univariate and multivariate Cox proportional hazard regression analysis to estimate the risk of death in patients diagnosed with locally advanced breast cancer. ER: estrogen receptor; HR: hazard ratio; PR: progesterone receptor; Ref: reference category. *Estrogen receptor (ER) and progesterone receptor (PR) status were combined as both negative (NN), both positive (PP), or only one positive (NP).

Characteristic	Univariate analysis				Multivariate analysis	
	Alive	Dead	HR	P-value	HR	P-value
	N = 120	N = 33				
Age						
<65	103 (82.4%)	22 (17.6%)	Ref	Ref	Ref	Ref
65+	17 (60.7%)	11 (39.3%)	2.08 (1.00-4.31)	0.049	3.20 (1.48-6.90)	0.003
Diagnosis						
Ductal	96 (78.7%)	26 (21.3%)	Ref	Ref		
Lobular	18 (90.0%)	2 (10.0%)	0.29 (0.07-1.24)	0.095		
Other	6 (54.5%)	5 (45.5%)	2.61 (1.00-6.85)	0.051		
T stage						
T1/2	74 (84.1%)	14 (15.9%)	Ref	Ref		
T3/4	46 (70.8%)	19 (29.2%)	2.22 (1.11-4.44)	0.025		
N stage						
N0/1	75 (86.2%)	12 (13.8%)	Ref	Ref		
N2/3	45 (68.2%)	21 (31.8%)	2.08 (1.02-4.23)	0.044		
ER/PR status*						
NN	24 (57.1%)	18 (42.9%)	Ref	Ref	Ref	Ref
NP	14 (70.0%)	6 (30.0%)	0.59 (0.23-1.50)	0.270	0.48 (0.19-1.22)	0.122
PP	82 (90.1%)	9 (9.89%)	0.17 (0.08-0.39)	<0.001	0.13 (0.05-0.30)	<0.001
HER2 status						
Negative	92 (77.3%)	27 (22.7%)	Ref	Ref		
Positive	28 (82.4%)	6 (17.6%)	1.15 (0.47-2.83)	0.765		
Targeted therapy						
No	96 (77.4%)	28 (22.6%)	Ref	Ref		
Yes	24 (82.8%)	5 (17.2%)	1.14 (0.43-3.04)	0.788		
Radiotherapy						
No	22 (68.8%)	10 (31.2%)	Ref	Ref		
Yes	98 (81.0%)	23 (19.0%)	0.69 (0.33-1.47)	0.343		
Chemotherapy						
None	16 (76.2%)	5 (23.8%)	Ref	Ref		
Adjuvant	66 (81.5%)	15 (18.5%)	0.65 (0.24-1.80)	0.407		
Neoadjuvant	38 (74.5%)	13 (25.5%)	1.61 (0.57-4.57)	0.373		
Type of breast surgery						
Lumpectomy	39 (86.7%)	6 (13.3%)	Ref	Ref	Ref	Ref
Mastectomy	81 (75.0%)	27 (25.0%)	2.76 (1.13-6.72)	0.026	2.44 (0.97-6.12)	0.05

**Figure 2:**
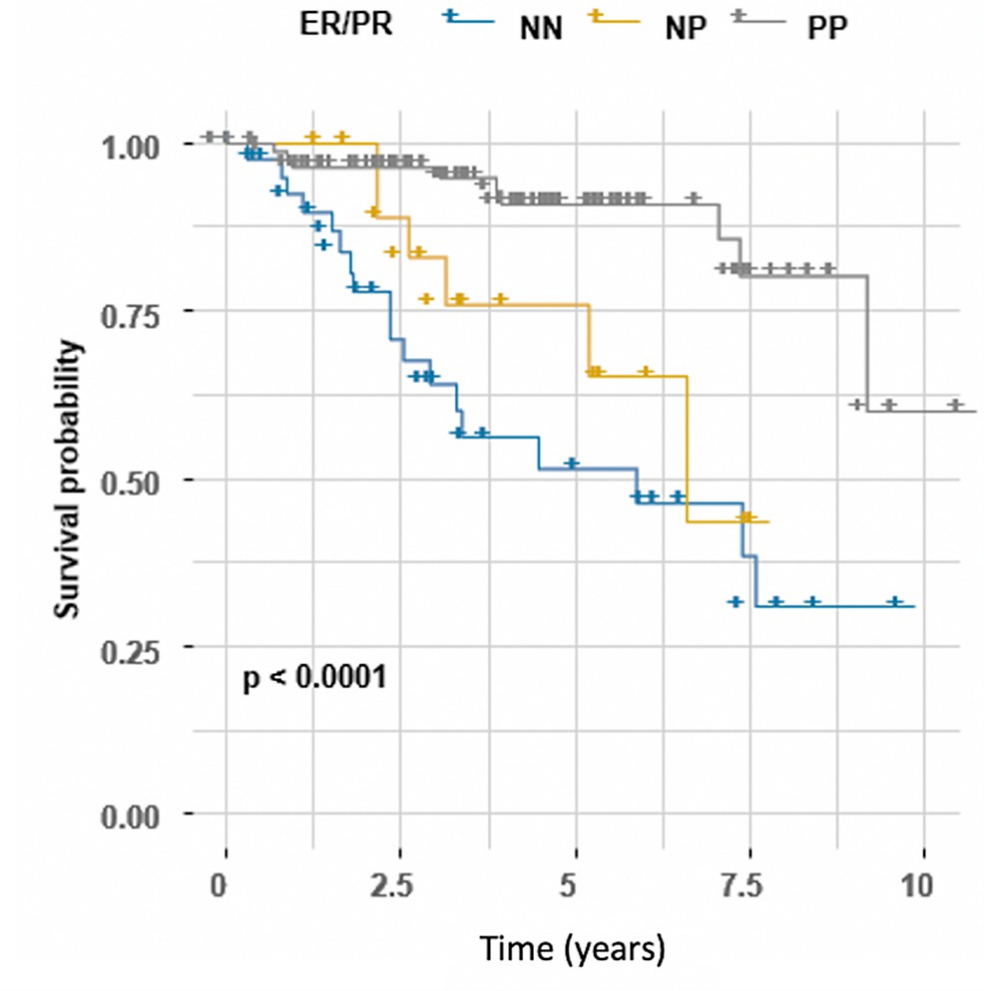
Kaplan-Meier estimator for estrogen (ER)/progesterone (PR) status, log-rank test (P < 0.0001). ER and PR status were combined as both negative (NN), both positive (PP), or only one positive (NP).

Having a tumor that expressed receptors for both ER and PR (PP) was associated with better OS than was having a tumor that expressed receptors for neither (NN) (HR 0.17, 95% CI 0.08-0.39, P < 0.001), whereas having a tumor that expressed receptors for only one (NP) did not influence survival status (Table 2). Survival curves across different groups are shown in Figure 2.

Higher T and N stages were associated with a higher hazard of death (HR 2.22, 95% CI 1.11-4.44, P = 0.025 and HR 2.08, 95% CI 1.02-4.23, P = 0.044, respectively) (Table 2).

**Figure 3:**
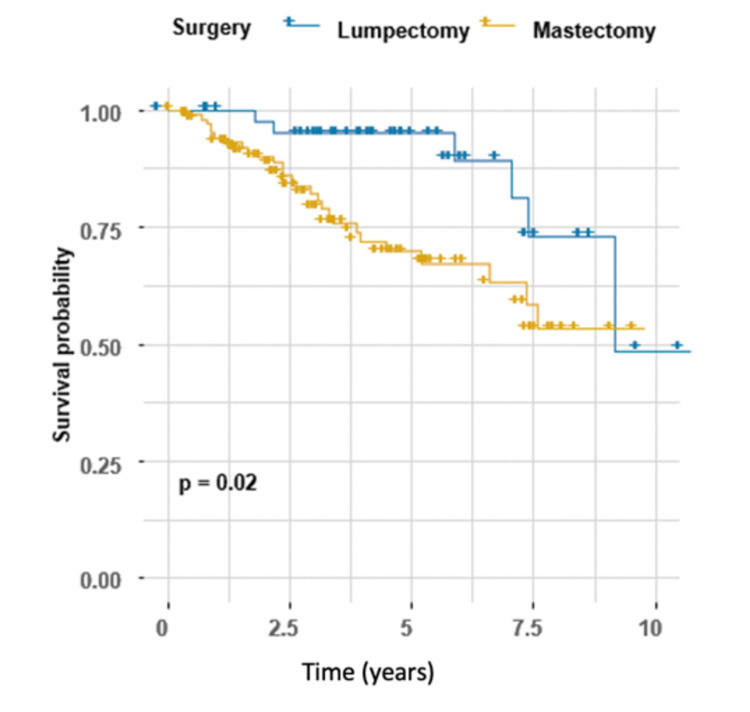
Kaplan-Meier estimator for surgery, log-rank test (P = 0.02).

Moreover, the hazard of death in patients who underwent mastectomy was significantly higher than in patients who underwent lumpectomy (HR 2.76, 95% CI 1.13-6.72, P = 0.026) (Table 2). Survival curves across both groups are shown in Figure 3.

HER2 status and treatment with chemotherapy, targeted therapy, or RT were not significantly associated with OS.

Multivariate Analysis

Multivariate CPH regression analysis was performed to estimate the risk of death in patients diagnosed with LABC. The final multivariate model is shown in Table 2. Age, receptor status, and type of breast surgery were predictors of death.

**Figure 4:**
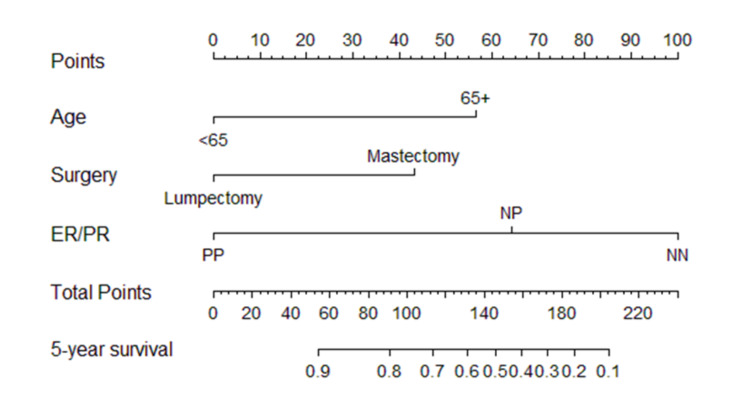
Validated nomogram to predict five-year survival of patients with locally advanced breast cancer. Estrogen receptor (ER) and progesterone receptor (PR) status were combined as both negative (NN), both positive (PP), or only one positive (NP).

The five-year survival prediction nomogram of the study population was validated by using 1,000 bootstrapped samples. The corrected Somers’ index (Dxy) was 58.41%, indicating a 58.41% conformity between the predicted and observed survival times. A patient who was <65 years at diagnosis and who had a lumpectomy of a tumor that expressed ER/PR (PP) had a greater than 90% probability of five-year survival (Figure 4).

Disease-free survival

Univariate Analysis

**Figure 5:**
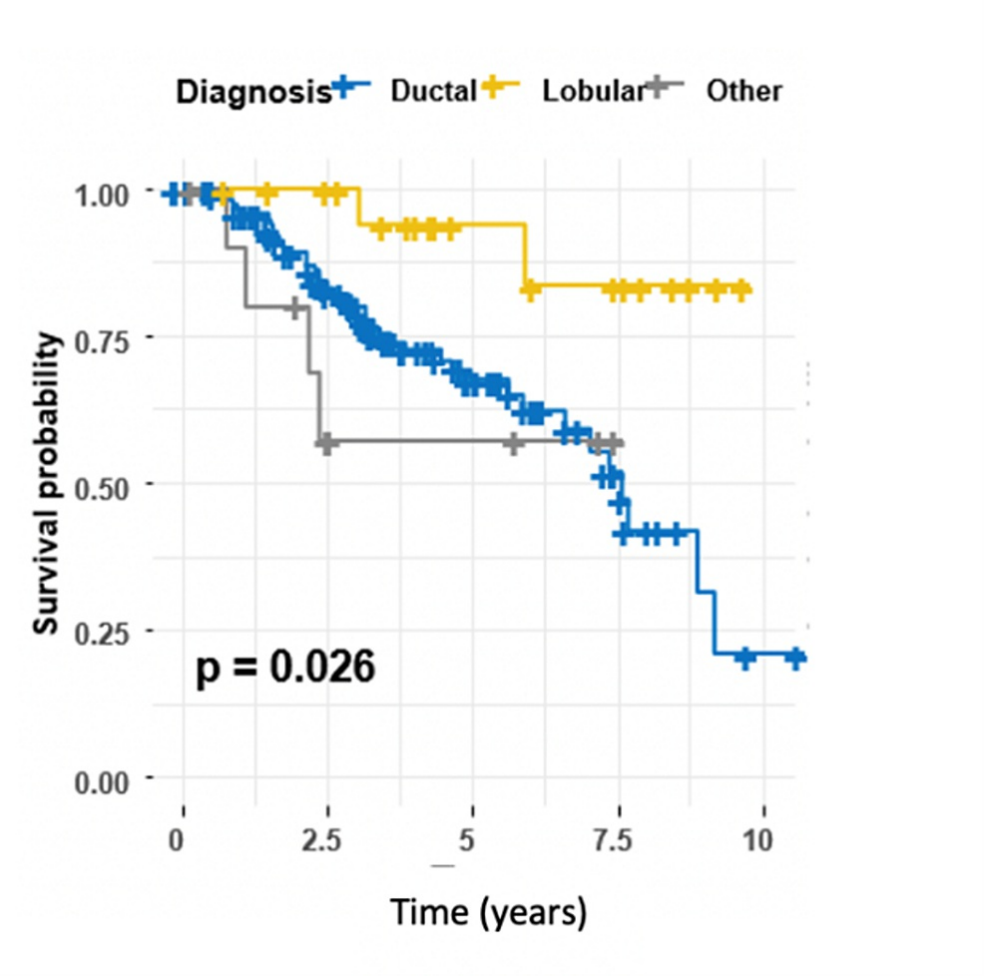
Kaplan-Meier estimator for the type of breast cancer, log-rank test (P = 0.026).

Our data showed that the type of BC, N stage, ER/PR status, and type of breast surgery were significant predictors for DFS, all of which were identified by using univariate CPH regression (Table 3). Lobular carcinoma was associated with a lower risk of recurrence than was ductal carcinoma (HR 0.19, 95% CI 0.05-0.81, P = 0.024) (Table 3). Survival curves across different pathological types of BC are shown in Figure 5.

**Table 3:** Univariate and multivariate Cox proportional hazard regression analysis to estimate the risk of disease-free survival in patients diagnosed with locally advanced breast cancer. ER: estrogen receptor; HR: hazard ratio; PR: progesterone receptor; Ref: reference category. *ER and PR status were combined as both negative (NN), both positive (PP), or only one positive (NP).

Characteristic	Univariate analysis				Multivariate analysis	
	No event	Event	HR	P-value	HR	P-value
	N = 108	N = 45				
Age						
<65	93 (74.4%)	32 (25.6%)	Ref	Ref	Ref	Ref
65+	15 (53.6%)	13 (46.4%)	1.64 (0.85-3.15)	0.140	2.21 (1.12-4.36)	0.022
Diagnosis						
Ductal	83 (68.0%)	39 (32.0%)	Ref	Ref		
Lobular	18 (90.0%)	2 (10.0%)	0.19 (0.05-0.81)	0.024		
Other	7 (63.6%)	4 (36.4%)	1.49 (0.53-4.21)	0.448		
T stage						
T1/2	66 (75.0%)	22 (25.0%)	Ref	Ref		
T3/4	42 (64.6%)	23 (35.4%)	1.76 (0.97-3.16)	0.061		
N stage						
N0/1	70 (80.5%)	17 (19.5%)	Ref	Ref		
N2/3	38 (57.6%)	28 (42.4%)	1.96 (1.07-3.59)	0.029		
ER/PR status*						
NN	21 (50.0%)	21 (50.0%)	Ref	Ref	Ref	Ref
NP	12 (60.0%)	8 (40.0%)	0.67 (0.30-1.52)	0.341	0.55 (0.24-1.26)	0.157
PP	75 (82.4%)	16 (17.6%)	0.26 (0.13-0.50)	<0.001	0.21 (0.11-0.41)	<0.001
HER2 status						
Negative	84 (70.6%)	35 (29.4%)	Ref	Ref		
Positive	24 (70.6%)	10 (29.4%)	1.49 (0.72-3.06)	0.280		
Targeted therapy						
No	87 (70.2%)	37 (29.8%)	Ref	Ref		
Yes	21 (72.4%)	8 (27.6%)	1.36 (0.62-2.99)	0.441		
Radiotherapy						
No	19 (59.4%)	13 (40.6%)	Ref	Ref		
Yes	89 (73.6%)	32 (26.4%)	0.72 (0.38-1.39)	0.331		
Chemotherapy						
None	14 (66.7%)	7 (33.3%)	Ref	Ref		
Adjuvant	66 (81.5%)	15 (18.5%)	0.45 (0.18-1.12)	0.086		
Neoadjuvant	28 (54.9%)	23 (45.1%)	1.98 (0.84-4.67)	0.119		
Type of breast surgery						
Lumpectomy	36 (80.0%)	9 (20.0%)	Ref	Ref	Ref	Ref
Mastectomy	72 (66.7%)	36 (33.3%)	2.54 (1.21-5.32)	0.013	2.41 (1.13-5.14)	0.023

**Figure 6:**
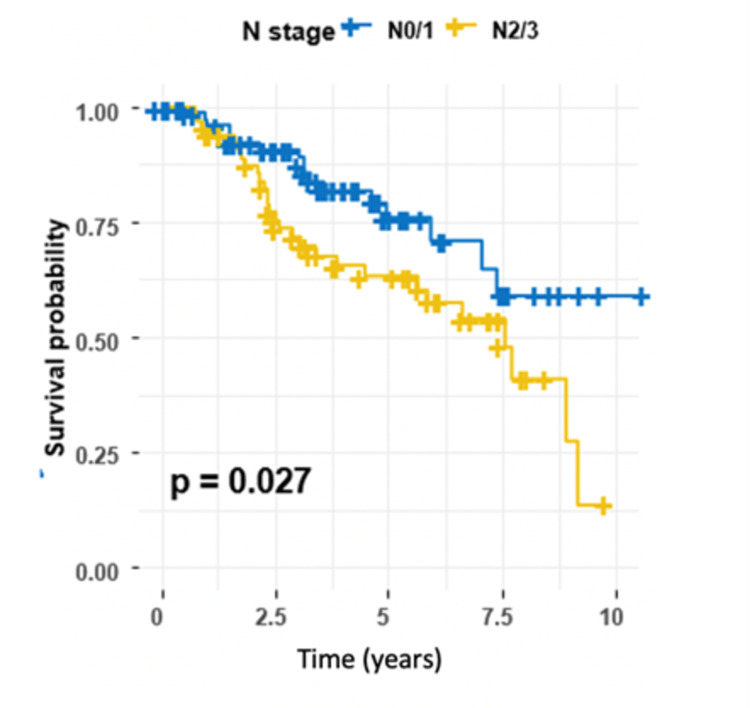
Kaplan-Meier estimator for N stage, log-rank test (P = 0.027).

Moreover, the results indicated that the risk of recurrence was twice as high in patients with N stage 2/3 as in those with N stage 0/1 (HR 1.96, 95% CI 1.07-3.59, P = 0.029) (Table 3). Survival curves across both groups are shown in Figure 6.

**Figure 7:**
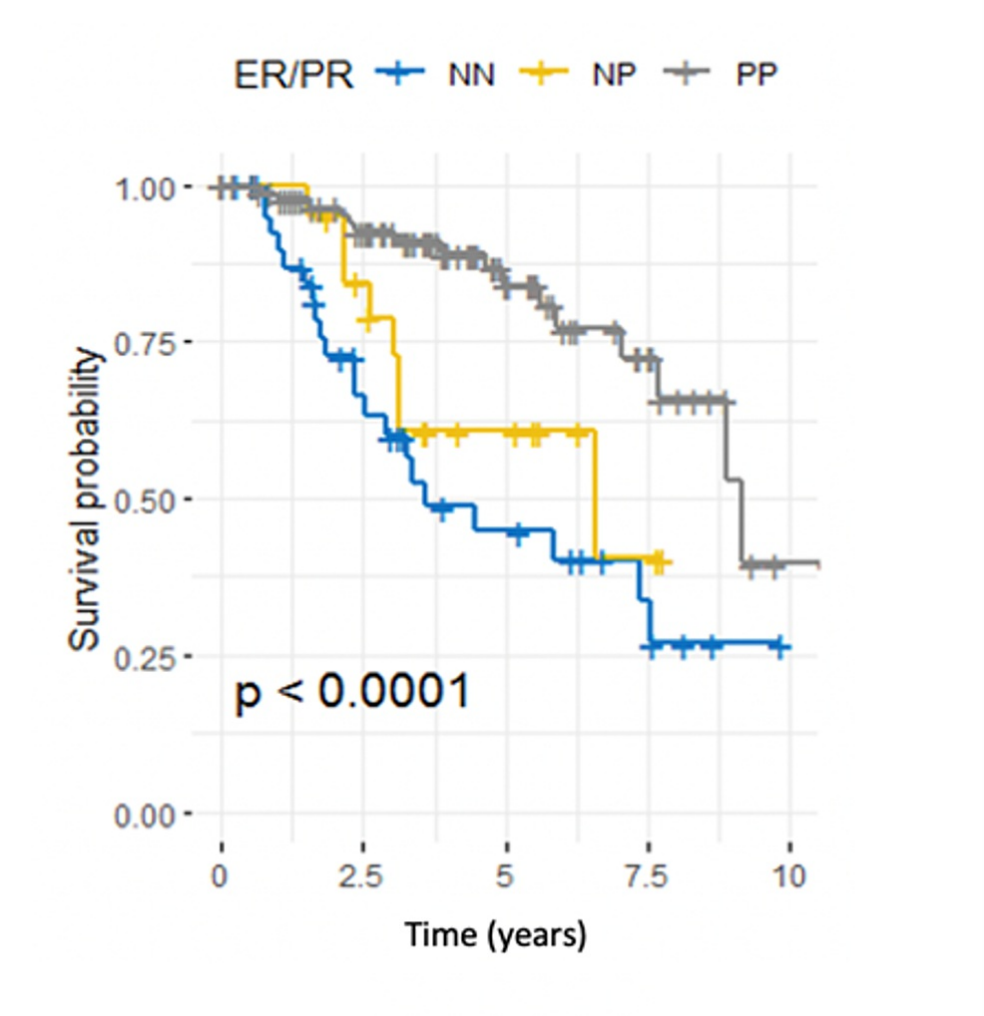
Kaplan-Meier estimator for estrogen receptor (ER)/progesterone receptor (PR) status, log-rank test (P < 0.001). ER and PR status were combined as both negative (NN), both positive (PP), or only one positive (NP).

Regarding receptor status, tumors that expressed both ER and PR (PP) were associated with a lower risk of recurrence than were those that expressed neither (NN) (HR 0.26, 95% CI 0.13-0.50, P < 0.001). However, expressing either ER or PR (NP) did not influence DFS (Table 3). Survival curves across different groups are shown in Figure 7.

**Figure 8:**
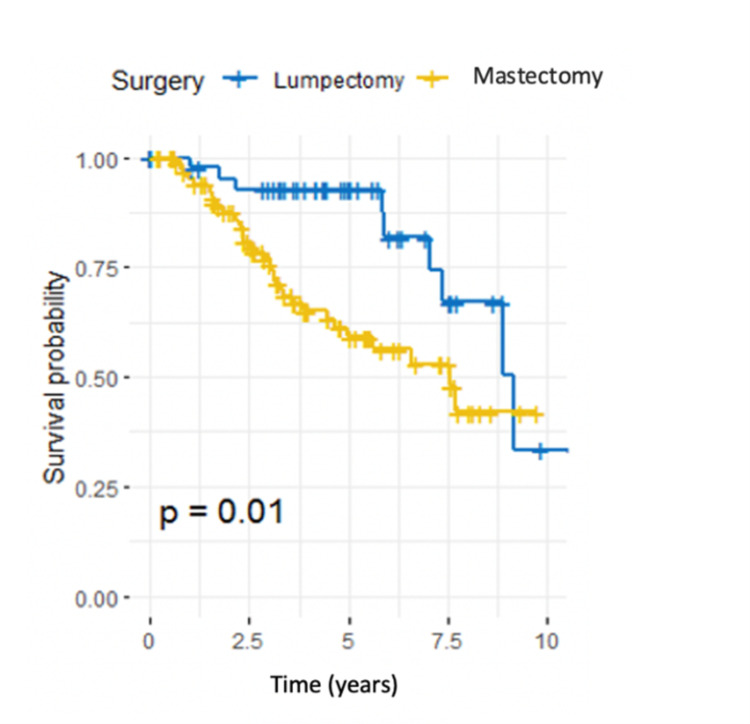
Kaplan-Meier estimator for the type of breast surgery, log-rank test (P = 0.01).

The risk of recurrence in patients who underwent mastectomy was significantly higher than in patients who underwent lumpectomy (HR 2.54, 95% CI 1.21-5.32, P = 0.013) (Table 3). Survival curves across both groups are shown in Figure 8.

HER2 status and treatment with chemotherapy, targeted therapy, or RT were not significantly associated with DFS.

Multivariate Analysis

Multivariate CPH regression analysis was performed to assess factors associated with disease relapse (Table 3). Age, receptor status, and type of breast surgery were predictors of disease relapse.

**Figure 9:**
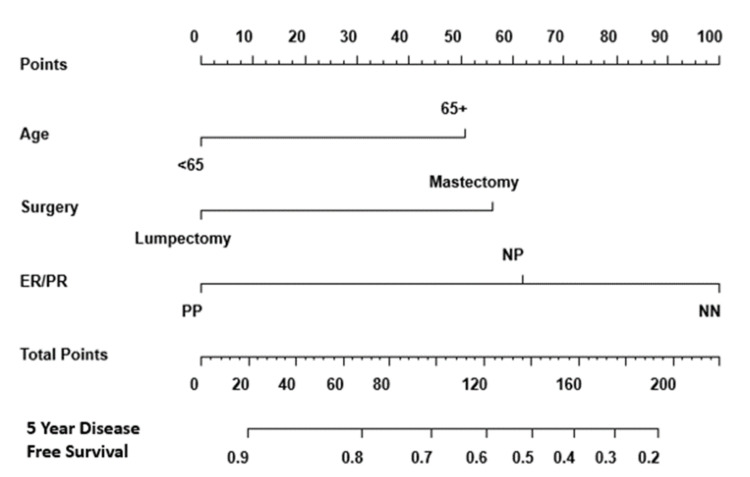
Validated nomogram to predict the probability of five-year disease-free survival for patients with locally advanced breast cancer. Estrogen receptor (ER) and progesterone receptor (PR) status were combined as both negative (NN), both positive (PP), or only one positive (NP).

The five-year DFS prediction nomogram of the study population was validated by using 1,000 bootstrapped samples. The corrected Somers’ index (Dxy) was 50.42%, which indicates a 50.42% concordance between the predicted and observed DFS times. A patient who was <65 years at diagnosis and who had a lumpectomy of a tumor that expressed ER and PR (PP) had a greater than 90% probability of five-year DFS (Figure 9).

Local recurrence and RT

Using the chi-square test of independence, we found that the risk of local recurrence was lower in patients with LABC who received RT than in those who did not (P = 0.011). The risk of recurrence was also associated with the technique used for RT (P = 0.003) (Table 4), as local recurrence occurred in 16.7% of patients who received step-and-shoot intensity-modulated RT (IMRT) and/or volumetric-modulated arc therapy (VMAT) but in 3.88% of patients who received three-dimensional conformal RT (3D-CRT). There was no statistically significant difference in the proportion of patients with local recurrence according to field number, dose, and whether or not they received a skin dose.

**Table 4:** Association of radiotherapy treatment and its related characteristics with local recurrence among patients diagnosed with locally advanced breast cancer. 3D-CRT: three-dimensional conformal radiotherapy; SSIMRT: step-and-shoot intensity-modulated radiotherapy; VMAT: volumetric-modulated arc therapy.

	Local recurrence		
	No	Yes	P-value
	N = 139	N = 14	
Radiotherapy			0.011
No	25 (78.1%)	7 (21.9%)	
Yes	114 (94.2%)	7 (5.79%)	
Technique			0.003
3D-CRT	99 (96.1%)	4 (3.88%)	
SSIMRT + VMAT	15 (83.3%)	3 (16.7%)	
Number of fields			0.592
0	18 (100%)	0 (0.0%)	
1, 2, and 3	96 (93.2%)	7 (6.80%)	
Skin dose			0.302
0 (bolus)	97 (95.1%)	5 (4.90%)	
1 and 2 (non-bolus)	17 (89.5%)	2 (10.5%)	
Dose			0.079
0	83 (95.4%)	4 (4.6%)	
1 and 2	22 (88%)	3 (12%)	

## Discussion

LABC constitutes the majority of BC cases in KSA and therefore creates a major burden socially, psychologically, and economically. Understanding the factors that affect survival in patients with LABC is of paramount importance [2].

Data in the literature about the impact of a patient’s age on the prognosis of BC are contradictory. Some authors reported that younger patients have a worse prognosis, whereas other authors reported elderly patients as having the worst prognosis [15-21]. Moreover, previous data in the United States, the United Kingdom, and KSA did not show any significant impact of patient age on OS and progression-free survival (PFS) in LABC [9,22,23]. These contradictory results could be attributed to the lack of fixed age cutoffs to define these groups (young vs elderly) among the studies.

A retrospective analysis published in 2017 was conducted on a cohort of 80 patients aged 70-96 years who were diagnosed with LABC in the Czech Republic. It showed that patients who were ≥80 years had a worse OS, with a median of 31.6 months (19.0-44.3) (HR 2.55, 95% CI 1.40-4.65, P = 0.002), than did patients who were <80 years, with a median of 78.5 months (51.4-105.5). Moreover, multivariate analysis also confirmed the significance of older age on OS in patients in the group who were ≥80 years (HR 4.76, 95% CI 1.22−18.61, P = 0.025) [17].

In our study, 81.7% of patients were younger than 65 years, and women older than 65 years represented only 18.3% of the cases. Our study also showed that OS and DFS in women who are over 65 years are worse than they are in women in a younger age group. Therefore, older age could be considered a negative prognostic factor in women with LABC.

Historically, upfront mastectomy was routinely performed in patients with LABC. The introduction of neoadjuvant systemic therapies has, however, increased interest in breast-conserving surgery (BCS) in LABC [24]. A prospective study with patients who underwent lumpectomy following neoadjuvant chemotherapy and postoperative RT showed an OS of 78% and a local recurrence rate of 6% at 91 months, which are acceptable rates [25]. In one review in which lumpectomy was performed in 33.24% of patients and mastectomy in 66.76%, the authors showed that the five-year DFS and OS for patients who received neoadjuvant chemotherapy and then underwent lumpectomy were 80.7% and 89.1%, respectively, and that DFS and OS for those who underwent mastectomy after neoadjuvant chemotherapy were 74.6% and 84.2%, respectively. There was no significant difference between the two groups (P = 0.9 and P = 0.217, respectively), which makes BCS an acceptable and safe option for patients with LABC after neoadjuvant chemotherapy [26].

A retrospective study that compared OS in patients with stage I-III BC who underwent BCS vs mastectomy showed that there was no significant difference between the two surgical approaches (HR 0.93, 95% CI 0.75-1.14, in the mastectomy group), irrespective of the adjuvant treatment received. However, the same study compared BCS and RT with mastectomy alone and revealed a significant survival benefit in the first group (HR 1.60, 95% CI 1.36-1.89, with mastectomy alone) [27].

On the other hand, a systematic review and meta-analysis published in 2017 examined 16 studies with a total of 3,531 patients, of whom 41.5% underwent BCS and 58.5% underwent mastectomy, to compare the outcome of surgery in patients with LABC who had a good response to neoadjuvant chemotherapy. The study showed a lower distant recurrence (odds ratio (OR) 0.51, 95% CI 0.42-0.63, P < 0.01), a higher DFS (OR 2.35, 95% CI 1.84-3.01, P < 0.01), and a higher OS (OR 2.12, 95% CI 1.51-2.98, P < 0.01) in the BCS group than in the mastectomy group. This improvement in survival can likely be attributed to the response to neoadjuvant treatment rather than the procedure itself. Furthermore, all patients in the BCS group received RT following surgery, whereas the mastectomy group included both those who received RT following surgery and those who did not. In addition, DFS and OS were mentioned in only some of the included articles, which may have led to high heterogeneity and affected the results of the study [28]. Nonetheless, this study suggests that survival rates are not related to the surgical approach alone, but also to the response to neoadjuvant treatment and RT.

In our study, 70.6% of patients underwent mastectomy and had worse OS and DFS than did patients who underwent lumpectomy for LABC. This result can likely be attributed to the nature of the aggressive disease and not the type of surgery, as this population included patients who had a poor response or who had disease progression during neoadjuvant treatment.

Regarding the role of luminal classification in LABC and its effect on OS and DFS, we found that hormone-positive disease was associated with better OS and DFS than was hormone-negative disease. This finding was concordant with the results of multiple previous studies. A large population-based study that included 4304 women compared the OS in hormone-positive disease (ER/PR PP) with that in hormone-negative disease (ER/PR NN). The two-year OS in patients with hormone-positive disease was 93% vs 78% in hormone-negative disease (P < 0.0001) [29].

Similar results were also observed in a prospective study that showed a significant difference in OS and PFS in favor of hormone-positive disease (ER/PR PP) (P < 0.01 and P < 0.01, respectively), with HER2/neu-enriched (ER/PR NN HER2+) and triple-negative disease (ER/PR NN HER2-) having the poorest OS and PFS [30]. This finding was further supported in other studies [31,32].

The role of targeted therapy in improving OS and DFS was not clearly demonstrated in our data, as there was a nonsignificant association. However, this might be attributed to the low prevalence of patients with HER2+ BC in our sample and the recent introduction of dual neoadjuvant anti-HER2 treatment at our institution. Nonetheless, other articles in the literature have shown that targeted therapy in patients with HER2+ BC improved OS in LABC [33,34].

The literature reported that patients with HER2+ disease who received neoadjuvant chemotherapy with HER2-targeted therapy and had residual disease had worse OS and DFS than did those who had a pathological complete response in early BC and LABC [33,35-38]. The current standard of care is to continue HER2-targeted therapy for one year +/- endocrine treatment for five years postoperatively [39].

Trastuzumab emtansine (T-DM1), an antibody-drug conjugate consisting of HER2-targeted therapy (trastuzumab) and a cytotoxic agent (DM1), is US Food and Drug Administration approved for patients with metastatic HER2+ disease who previously received treatment with trastuzumab and taxane, as it prolongs OS and PFS with a lower toxicity profile than does a capecitabine plus lapatinib regimen [40-44]. In 2019, a phase 3 open-label trial was conducted on post-neoadjuvant treatment with trastuzumab and taxane that included patients with non-metastatic HER2+ disease who had residual invasive disease at surgery. The patients were randomized into adjuvant T-DM1 and adjuvant trastuzumab-alone groups. Compared with trastuzumab alone, T-DM1 as adjuvant treatment was found to improve OS (HR for death 0.70, 95% CI 0.47-1.05, P = 0.08) and invasive DFS (HR for disease recurrence or death 0.50, CI 0.39-0.64, P < 0.001) [45].

RT is an integral component in the management of LABC. In operable LABC, the addition of adjuvant RT improves locoregional control and OS in stage III disease [46]. Even in the setting of neoadjuvant chemotherapy, postmastectomy RT was associated with a significantly reduced locoregional recurrence rate at 10 years for all patients, but more so for patients with stage III or IV disease who achieved a pathological complete response (33% vs 3% at 10 years, P = 0.006) [12].

The Early Breast Cancer Trialists' Collaborative Group (EBCTCG) meta-analysis that included 8135 patients revealed that RT decreases both BC recurrence and mortality [47]. Multiple other trials, including the MA.20, the EORTC 22922-10925, and the French trial, concluded that regional RT correlated with a significant further increase in PFS (HR 0.85) and OS (HR 0.83) [48,49].

Adjuvant RT in our cohort, of whom more than half had stage T1-T2 and/or N0-1 disease, was associated with improved local control, but not with DFS or OS. A possible survival advantage in patients with more advanced stages and/or certain molecular subtypes could not be elicited because of the small sample size. A large retrospective study of 8,935 patients in Germany revealed that when RT was given in accordance with international guidelines (after mastectomy for T3-T4 or N2 disease), it was associated with improved OS [50].

Multiple authors have also found that RT improves survival for women with early triple-negative BC [51-53]. Others, however, have reported conflicting results [54,55]. In the only randomized trial to have addressed this issue, Wang et al. [52] revealed survival advantage from the addition of RT in triple-negative BC. There has therefore been growing interest in treatment escalation for women with triple-negative BC.

On the other hand, women with luminal A (ER/PR PP HER2-) or hormone-positive/HER2+ (ER/PR PP HER2+) BC are thought to have very low risk of locoregional recurrence. The following approaches are therefore considered reasonable for these groups and are subject to ongoing trials: (1) for luminal A BC, de-escalating treatment by omitting RT for node-negative disease after lumpectomy for older women or after mastectomy with limited nodal involvement; (2) for hormone-positive/HER2+ BC, omitting RT for complete responders to neoadjuvant chemotherapy [56-59]. Incorporating gene assays in RT decision-making is another area of growing interest [59,60].

Although the local control benefit of adjuvant RT has been well documented in the literature [47,61,62], the differential effect of different technical aspects of RT has not been well studied. Our study showed that 3D-CRT was associated with local control that was superior to that of IMRT/VMAT. This could simply be a reflection of more advanced disease, such as internal mammary involvement, necessitating more complex treatment, but it could also indicate a potential geographical target miss with IMRT/VMAT. A precise definition of clinical targets and a careful assessment of the needed safety margin are crucial. The variability in target delineation in most tumor sites has been associated with significant uncertainty in the RT treatment process and subsequent dose delivery, which could potentially affect local control [63,64].

The number of fields treated, which indicates breast/chest wall only vs locoregional treatment, and the use of a bolus to increase the skin dose was not associated with a difference in local control in our cohort. In accordance with the available literature, hypofractionation and standard fractionation schedules were equivalent in terms of local control [65-67].

Study limitations

Our study is one of the few to assess the outcomes and predictors of LABC in our region. Nonetheless, one of the main limitations is that it is a retrospective study, and the relatively small sample size could have affected the identification of significant predictors. As with all retrospective studies, loss of follow-up is a limiting factor; however, we maximized efforts to reach out to patients who did not come for follow-up in the last two years in order to identify those who had a recurrence, who died, or who were treated elsewhere.

## Conclusions

Multiple factors can affect the OS and DFS in LABC. Younger patients, having hormone-positive disease, and undergoing a lumpectomy were associated with better outcomes. Adjuvant RT may improve local control and the use of 3D-CRT was a superior modality in terms of local control. However, prospective studies with larger sample sizes are needed to further highlight these findings and to assess the role of chemotherapy and targeted therapy in patients with LABC.
